# A wish list for synthetic biology in photosynthesis research

**DOI:** 10.1093/jxb/eraa075

**Published:** 2020-02-15

**Authors:** Xin-Guang Zhu, Donald R Ort, Martin A J Parry, Susanne von Caemmerer

**Affiliations:** 1 Institute of Plant Physiology and Ecology and Center for Excellence for Molecular Plant Sciences, Chinese Academy of Sciences, Shanghai, China; 2 Departments of Plant Biology and Crop Sciences, University of Illinois at Urbana Champaign, Urbana, IL, USA; 3 Lancaster Environment Centre, Lancaster University, Lancaster, UK; 4 Research School of Biological Sciences, Australian National University, Acton, Australia; 5 MPI of Molecular Plant Physiology, Germany

**Keywords:** Artificial photosynthesis, carboxysome, C_4_ engineering, fluorescence marker proteins, natural variation, photosynthesis, synthetic biology

## Abstract

This perspective summarizes the presentations and discussions at the ‘ International Symposium on Synthetic Biology in Photosynthesis Research’, which was held in Shanghai in 2018. Leveraging the current advanced understanding of photosynthetic systems, the symposium brain-stormed about the redesign and engineering of photosynthetic systems for translational goals and evaluated available new technologies/tools for synthetic biology as well as technological obstacles and new tools that would be needed to overcome them. Four major research areas for redesigning photosynthesis were identified: (i) mining natural variations of photosynthesis; (ii) coordinating photosynthesis with pathways utilizing photosynthate; (iii) reconstruction of highly efficient photosynthetic systems in non-host species; and (iv) development of new photosynthetic systems that do not exist in nature. To expedite photosynthesis synthetic biology research, an array of new technologies and community resources need to be developed, which include expanded modelling capacities, molecular engineering toolboxes, model species, and phenotyping tools.

## Teaser

To expedite advances in synthetic biology in photosynthesis research, new technologies and community resources need to be developed.  These include expanded modelling capacities, molecular engineering toolboxes, model species, and phenotyping tools.

## Introduction

Photosynthesis provides the basis for food, feed, and fibre for humanity and is a critical central component of the global ecosystem. Improving photosynthetic efficiency has recently emerged as a key strategy to increase yield potential of major crops. Synthetic biology in photosynthesis is an emerging new research field which aims to develop new parts, pathways, and regulatory circuits under the guidance of systems modelling with the goal of creating more efficient photosynthetic systems. Research in this field spans a wide scope from the optimization of existing photosynthetic systems to the creation of altogether new photosynthetic systems or component processes that do not exist in nature.

This perspective on new opportunities to redesign and engineer photosynthesis arose out of discussions held at the ‘International Symposium on Synthetic Biology in Photosynthesis Research’ which took place in Shanghai in August 2018. Two days of talks and poster presentations by a broad spectrum of international experts informed the symposium’s culminating discussion about what might be achieved, what the priorities should be for the greatest impact, what are the greatest obstacles, and what sorts of new tools are needed to overcome them. Here we report the major outcomes of the symposium’s concluding synthesis session. Our intent (summarized in Table 1) is an updated look at new opportunities to enhance the photosynthetic process for targeted applications employing new tools and innovations emerging in synthetic biology.

**Table 1.** The opportunities for photosynthesis synthetic biology research and major challenges

Categories of photosynthesis engineering	Engineering options	Major challenges	Literature
Optimization and engineering of existing photosynthetic systems	Optimizing Rubisco kinetic properties	Chloroplast transformation in major crops	Sharwood (2017)
	Increasing Rubisco activation speed	No	Taylor and Long (2017)
	Optimizing the structure of ATP synthase	Chloroplast transformation in major crops	No^*a*^
	Antenna size of photosystems	No	Ort and Melis (2011); Song *et al.* (2017)
	Optimization of carbon metabolism enzymes	No	(Zhu *et al.* (2007); Simkin *et al.* (2015)
	Optimizing leaf anatomy	Identifying optimal leaf anatomical features for photosynthetic efficiency	Lundgren *et al.* (2019)
	Optimizing speed of non-photochemical quenching relaxation during light switching	No	Kromdijk *et al.* (2016)
Optimization of the interaction between photosynthesis and other processes utilizing photosynthate	Optimization of photosynthate transport, storage, and utilization	Identify limiting factors controlling source, sink, and flow	Chang *et al.* (2017)
	Releasing the feedback inhibition of photosynthate to photosynthesis	Elucidate the molecular basis of inhibition of photosynthesis by photosynthate	Chang *et al.* (2017)
	Engineering response of photosynthesis to phytohormones	Elucidate the molecular basis controlling responses of photosynthesis to phytohormones	Gururani *et al.* (2015)
	Engineering plant primary metabolism to enhance photosynthesis	Elucidate the interaction between photosynthesis, respiration and nitrogen assimilation	No^*b*^
Reconstruction of existing high-efficiency systems into current C_3_ crops	Engineering C_4_ CO_2_-concentrating mechanism into C_3_ leaves	Elucidate the molecular basis controlling Kranz anatomy	von Caemmerer *et al.* (2012); Sedelnikova *et al.* (2018)
	Engineering carboxysome based CO_2_-concentrating mechanism into C_3_ mesophyll cells	Elucidate the major elements of carboxysome and chloroplast transformation in crops	Price *et al.* (2011, 2013); McGrath and Long (2014)
	Engineering pyranoid into chloroplasts of C_3_ crops	Elucidate the major components of pyranoid and chloroplast transformation in crops	Mackinder *et al.* (2017)
	Engineering crassulacean acid metabolism (CAM) into chloroplasts of C_3_ crops	Elucidate the molecular basis controlling CAM formation	Yang *et al.* (2015)
	Developing phycobillisome	Elucidate the minimal elements needed for phycobillisome construction	Tang *et al.* (2015); Zhang *et al.* (2017); Zhao *et al.* (2017)
	Creation of the chlorophyll *d* and *f* pathway	Elucidate the metabolic basis for chlorophyll *d* and *f* synthesis	Blankenship and Chen (2013); Li and Chen (2015)
Creation of new photosynthetic systems that do not exist in nature	Creation of photorespiratory bypass pathway	No	Kebeish *et al.* (2007); Xin *et al.* (2015); Shen *et al.* (2019); South *et al.* (2019)
	Creation of new CO_2_ fixation pathway	Chloroplast transformation in crops	Bar-Even *et al.* (2010); Shih *et al.* (2014)
	Creation of autotrophic *E. coli*	No	Antonovsky *et al.* (2016)
	Creation of new pathway to utilize cellulose for production of high-value product	Pathway design and evolution-guided engineering strategy	Ji *et al.* (2012)
	Utilizing another energy source to support CO_2_ fixation	Pathway design	Gleizer *et al.* (2019)
Develop composite systems by combining photosynthesis and artificial material	Artificial photosynthesis	Material development	Zhang *et al.* (2015); Honda *et al.* (2016); Sakimoto *et al.* (2016)
	Creation of new material to enable better utilization of light and CO_2_	Develop artificial material to increase light absorption or increase intercellular CO_2_ concentration	No^*c*^

^*a*^ The number of helical proteins in the intramembrane F_0_ complex of the ATP synthase differs between species, suggesting that there might be an optimal number of helical proteins required to gain the maximal light use efficiency in plants.

^*b*^ Respiration, photorespiration, and nitrogen assimilation interact closely with photosynthesis, and greatly influence the efficiency of photosynthesis, such as the increase of photosynthetic CO_2_ uptake rate under photorespiratory conditions when NO_3_^–^ was supplied as a nitrogen source (Busch *et al.*, 2018), and the supply of α-ketoglutarate by the citric acid pathway to support ammonia assimilation (Sweetlove *et al.*, 2010). Optimization of photosynthesis requires better understanding of the interaction between photosynthesis, nitrogen assimilation, and respiration, and correspondingly coordination of photosynthesis with these closely interacting processes.

^*c*^ A large fraction of incident solar energy is outside of the photosynthetic active radiation spectrum. Development of synthetic material which can convert these photons into photons that can be utilized by photosynthesis is one direction that needs be explored (Boriskina and Chen, 2014). Similarly, synthetic material capable of increasing leaf intercellular CO_2_ concentration is another area in which synthetic chemistry techniques can be used to promote photosynthesis.

## Optimization of natural photosynthetic systems

Though photosynthesis has evolved over billions of years, the resultant photosynthetic processes are not fully optimized for the conditions of agricultural food/feed production and have been unable to keep up with the rapid pace of current ongoing environmental change. Therefore, identifying steps or components where current photosynthetic systems are suboptimal under different conditions and then optimizing these steps for optimal performance represents a major research thrust in current photosynthetic improvement efforts (Long *et al.*, 2015). Processes or properties that might be optimized for improved photosynthetic performance of agricultural crops include, but are not limited to, Rubisco kinetic properties, the structure of ATP synthase, antenna size of photosystems, abundance and activation state of the different enzymes in the photosynthetic carbon metabolism, leaf structure, and the response of photosynthesis to fluctuating light (Table 1).

A specific opportunity highlighted in the symposium focuses on the limitations to CO_2_ diffusion within leaves and across cell wall and membrane interfaces that perforce lower CO_2_ partial pressure at the sites of carboxylation in the chloroplast of C_3_ species and the mesophyll cytosol of C_4_ species (Evans and von Caemmerer, 1996; Flexas *et al.*, 2013). Enhancements of photosynthesis through manipulation of chloroplast function will be diminished through reduction in chloroplast CO_2_ partial pressure unless it is combined with improved internal CO_2_ diffusion properties of leaves. Methods to measure mesophyll conductance to CO_2_ diffusion require stable isotope techniques such as the fractionation of ^13^C or ^18^O in CO_2_ during CO_2_ assimilation, or combined measurement of chlorophyll fluorescence and CO_2_ assimilation, or using the signal of ^18^O-labelled water fed in the transpiration stream (Pons *et al.*, 2009; Gauthier *et al.*, 2018). For C_4_ species, only the ^18^O isotope technique can be used (Barbour *et al.*, 2016; Ubierna *et al.*, 2017). This difficulty in measuring CO_2_ conductance within leaves has hampered progress in the mechanistic understanding of determinants of mesophyll conductance and has hampered screening for genotypes with improved CO_2_ diffusion properties. It has been demonstrated that mesophyll conductance in C_3_ and C_4_ species is dynamic and increases with temperature, which is most probably due to changes in plasma membrane and chloroplast envelope membrane properties, and may involve aquaporins, although the processes that contribute to the temperature dependence are not well understood (von Caemmerer and Evans, 2015; Groszmann *et al.*, 2017; Ubierna *et al.*, 2017). Recent insights from barley mutants show that not only cell wall thickness but also their composition affects CO_2_ diffusion (Evans *et al.*, 2009; Ellsworth *et al.*, 2018). Mutant screening with combined measurements of leaf temperature and chlorophyll fluorescence is one way forward (McAusland *et al.*, 2013), as are the development of other imaging technologies for assessing leaf anatomy and cell wall properties.

Work to optimize Rubisco kinetic properties and regulatory mechanisms was also highlighted (Parry *et al.*, 2013). Improving the performance of Rubisco by protein engineering is a critical strategy for increasing crop yields. However, the extensive chaperone requirement of higher plant Rubisco for folding and assembly has long been an impediment to this goal. Recently, expressing higher plant Rubisco together with the essential chaperoning proteins successfully resulted in the synthesis and assembly of functional Rubisco in *Escherichia coli* (Wilson *et al.*, 2019), which will provide new opportunities for Rubisco engineering and optimization, possibly enabling directed evolution.

## Coordination of photosynthesis with the pathways utilizing photosynthate

There are fine-tuned interactions between photosynthesis and downstream processes utilizing photosynthate that must be considered and accommodated in synthetic redesigns. Photosynthate must be effectively utilized not only to sustain high photosynthetic efficiency by releasing metabolic feedback inhibition and genetic down-regulation, but also to maximize the supply of substrate for production of high-value agricultural and industrial products. There is a wide array of untapped opportunities to manipulate source–sink interactions to optimize photosynthate transport, storage, and consumption with the goal of increasing photosynthetic light use efficiency during the whole growing season. Creating new and/or longer duration sinks stands out as an engineering goal with the promise of near-term outcomes.

In the bioenergy sector, biomass requires significant onward processing to obtain liquid fuels, whereas higher value bioproducts such as triacyl glycerides may be easily and cost-effectively converted to biodiesel, or, with more processing, to jet fuel, lubricants, and products parallel to many produced from fossil fuel oil (Chernova *et al.*, 2010). Biosynthetic strategies to recircuit the flow of fixed carbon from carbohydrate into higher energy density oils could reduce energy crop biomass production by redirecting energy away from growth, which in turn can limit oil yield. The opportunity also exists to engineer high-value crops for fodder and food through increased oil production in leaves and seed (Vanhercke *et al.*, 2019; Zhou *et al.*, 2019). The trade-off between oil production and growth could be overcome by coupling the re-circuiting of carbon flow into oil to increasing photosynthetic efficiency of energy crops, allowing for enhanced oil production without a growth penalty.

Ever advancing *in silico* models of photosynthesis integrated into biophysical representations of the crop canopy can be used to reveal the best targets for synthetic improvement of photosynthetic performance in C_4_ bioenergy grasses. The engineering of C_4_ grasses has been significantly advanced with the development of *Setaria viridis* as a model genetic and genomic system (Li and Brutnell, 2011). A large number of new enabling genetic and genomic tools, methods, and resources are now available to the *S. viridis* community (Bennetzen *et al.*, 2012; Huang and Brutnell, 2016).

## Reconstruction of highly efficient photosynthetic systems in non-host species

In nature, to ensure survival in environments where access to either CO_2_ or light is frequently limited, different forms of photosynthetic systems with high CO_2_ and light use efficiency have evolved. Reconstruction of these different photosynthetic systems into crops represents a major focus of current photosynthesis synthetic biology research. A long-term strategy to enhance global crop photosynthesis and yield involves the introduction of cyanobacterial CO_2_-concentrating mechanisms (CCMs), such as the carboxysome-based cyanobacterial CCM, the pyranoid-based CCM, along with the required bicarbonate and/or CO_2_ transporters (Long *et al.*, 2016), into plant chloroplasts. An equally expansive long-term aspiration is the extension of the light absorption spectrum by current crops. The light absorption spectrum of land plants might be expanded by utilizing phycobilisomes or developing pathways for the incorporation of chlorophyll *d* and *f* (Blankenship and Chen, 2013; Li and Chen, 2015).

Among the most exciting new results reported at the symposium was evidence for the first ever assembly of a cyanobacterial α-carboxysome structure in higher plant chloroplasts (Long *et al.*, 2018). Cyanobacterial CCMs enable efficient CO_2_ fixation by elevating intracellular bicarbonate, then concentrating it as CO_2_ around Rubisco in protein micro-compartments called carboxysomes. While a fully functional carboxysome requires coordinated expression of nearly a dozen proteins, a simplified carboxysome has now been expressed in tobacco chloroplasts that enables autotrophic growth at elevated CO_2_, setting the stage for installing a fully functioning carboxysome in plant chloroplasts. Earlier, the β-carboxysome component CcmM35 was successfully transgenically integrated into tobacco together with the Rubisco large and small subunit from the cyanobacterium *Synechococcus elongatus* PCC7942 (Se7942) (Lin *et al.*, 2014). These proteins were shown to form macromolecular complexes in chloroplast stroma, also mimicking an early step of carboxysome formation. Ttransiently expressing multiple β-carboxysomal proteins including CcmK2, CcmM, CcmL, CcmO, and CcmN in *Nicotiana benthamiana* resulted in highly organized assembly mimicking an empty compartment (Lin *et al.*, 2014). The SSUL module of the CcmM is involved in the aggregation of Rubisco, which is required for carboxysome biogenesis (Wang *et al.*, 2019).

## Development of new photosynthetic systems that do not exist in nature

Creation of photosynthetic systems that do not exist in nature is synthetic biology in its truest form. Because the low substrate specificity and catalytic turnover of Rubisco are causal to the underperformance of photosynthesis, new pathways for CO_2_ fixation without utilization of the nitrogen-costly and inefficient Rubisco are being designed (Bar-Even *et al.*, 2010; Shih *et al.*, 2014). An autotrophic *E. coli* has been developed, which can be used as a testing chassis to expedite photosynthetic pathway design and engineering (Antonovsky *et al.*, 2016)

A convincing example of the power of introducing new pathways into photosynthesis presented at the symposium was the introduction of synthetic pathways to shortcut photorespiration. Photorespiration is required in C_3_ plants to metabolize inhibitory phosphoglycolate formed when Rubisco oxygenates rather than carboxylates ribulose-1,5-bisphosphate. Depending on growing temperatures, photorespiration can reduce yields by 20–50% in C_3_ crops (Walker *et al.*, 2016). Building on earlier work (Kebeish *et al.*, 2007), synthetic glycolate metabolic pathways that are thought to be more efficient than the native pathway were installed in tobacco chloroplasts (South *et al.*, 2019). Flux through the synthetic pathways can be maximized by strongly suppressing glycolate export from the chloroplast. The synthetic pathways tested improved photosynthetic quantum yield by 20%. Numerous homozygous transgenic lines increased biomass productivity by >19–37% in replicated field trials.

## New modelling capacities

Systems models play a crucial role in evaluating the major bottlenecks of current photosynthetic systems, and in design of new metabolic pathways and a signalling circuit to improve photosynthetic efficiency. Systems models at cellular, leaf, whole-plant, and canopy scales are needed to fully allow systematic evaluation of potential new options to improve photosynthesis. A new reaction diffusion model of C_3_ leaf photosynthesis has been developed, which allows evaluation of the impacts of manipulation of different leaf anatomical and metabolic features on light environments and CO_2_ concentration inside a leaf, which will facilitate evaluation of the effects of these manipulations on mesophyll conductance and photosynthetic efficiency for a leaf (Xiao *et al.*, 2016; Xiao and Zhu, 2017).

New modelling was also reported that enabled systematic evaluation of the coordination between metabolic and anatomical features revealing that NADP-ME and NAD-ME C_4_ types have different obligate anatomical features (S Wang *et al*., unpublished) as well as revealing the metabolic basis for C_4_ chloroplast dimorphism (Bräutigam *et al.*, 2018, Preprint). Canopy-scale modelling suggests that slow induction of photosynthesis on shade to sun transition may decrease crop productivity by up to 21% (Taylor and Long, 2017). With a cross-scale model, the yield consequences of different options of increasing photosynthetic capacity were evaluated to find that the yield responses vary greatly and depend heavily on photosynthetic enhancement, crop type, and environment (Wu *et al.*, 2019). The study also highlighted that better understanding and modelling of photosynthesis–stomata linkage are required to gain accurate prediction of future crop yield responses to transgenic manipulations. It has become clear that the dynamic behaviour of stomatal responses to light influences photosynthesis, biomass accumulations, and water use efficiency (McAusland *et al.*, 2016). Innovative research overexpressing a synthetic blue light-activated K^+^ channel has demonstrated that stomatal opening and closing can be accelerated to increase biomass production (Papanatsiou *et al.*, 2019).

## Development of major tools that are needed to support photosynthesis synthetic biology research

Photosynthesis synthetic biology research is highly interdisciplinary, where progress is frequently constrained by available technology. To implement synthetic redesigns of the photosynthetic process effectively, many new tools and platforms need to be developed and shared across the community. Here we briefly synthesize the major tools and platforms identified from the symposium.

(i)* Molecular toolset for genetic engineering*: including efficient gene editing systems for targeted engineering of single or multiple target genes for either knockout of, increasing, or decreasing expression; a repertoire of promoters with high tissue specificity, developmental stage specificity, and different expression strengths; and efficient chloroplast transformation systems for crop plant species.
(ii)* Model systems*: including photosynthetically active suspension cell lines, different chasses for photosynthesis synthetic biology encompassing different algae, Arabidopsis, *Setaria*, maize, and plants for production of high-value products.
(iii)* Techniques to measure different photosynthetic properties at multiple scales*: development of highly versatile techniques to enable systematic measurement of photosynthetic properties, including biophysical, biochemical, and physiological parameters at the molecular, cellular, leaf, and canopy scales; development of techniques to enable *in situ*, ideally 3D, detection and visualization of the water, minerals, and metabolites at different spatial scales and time resolutions. On this aspect, genetically encoded fluorescence sensors developed to obtain *in vivo* measurement of metabolite concentrations (Zhang *et al.*, 2011; Lindenburg and Merkx, 2014; Zhao *et al.*, 2015; Tao *et al.*, 2017), or subcellular localization of proteins (Tanz *et al.*, 2013) and new algorithms to enable ^13^C labelling-based metabolite flux analysis for autotrophic cells and tissues (Ma *et al.*, 2014; Arrivault *et al.*, 2017; Zhang *et al.*, 2018) are beginning to emerge and have the potential to become powerful new tools.
iv ) *Development of a new theoretical framework to support systems design and engineering*: development of systems models of photosynthesis covering multiple scales, spanning from molecular, organelle, cellular, tissue, organ, plant, canop, and up to ecosystem scales; development of methods for model construction, parameterization, validation, and optimization; improvement of the theory for control analysis of complex models (see perspective in Xiao and Zhu, 2017).
v)* Development of online research resources to support photosynthesis synthetic biology research*: a centralized research portal to support photosynthesis synthetic biology research may include a system to order major molecular tools to support genetic engineering, a database of the major engineering design options and their impacts on photosynthesis in different model systems; systems modelling and designing function to support online design and data analysis; standard protocols for both genetic engineering and phenotyping (Patron *et al.*, 2015; Pollak *et al.*, 2019); and a forum to support discussions among photosynthesis synthetic biologists.

## Concluding remarks

The field of synthetic biology in photosynthesis research has been highly cross-disciplinary from its beginning. It not only involves research from the traditional biophysics, biochemistry, molecular biology, and physiology of photosynthesis, but also engages with research in the field of systems modelling, genome editing, guided evolution, and novel phenomics technologies. We created this symposium to serve as a meeting venue to support exchange of ideas in this exciting field, which promises to deliver solutions to many of the contemporary challenges facing our society.
